# First-line pembrolizumab plus chemotherapy versus chemotherapy alone for advanced esophageal cancer: 5-year extended follow-up in the Japanese subgroup of KEYNOTE-590

**DOI:** 10.1007/s10388-026-01200-8

**Published:** 2026-04-28

**Authors:** Ken Kato, Takashi Kojima, Hiroki Hara, Akihito Tsuji, Hisateru Yasui, Kei Muro, Taroh Satoh, Naoyoshi Yatsuzuka, Tomoko Sakata, Shirong Han, Toshihiko Doi

**Affiliations:** 1 Department of Head and Neck, Esophageal Medical Oncology, National Cancer Center Hospital, 5-1-1 Tsukiji, Chuo-Ku, Tokyo, 104-0045 Japan; 2 Department of Gastroenterology and Gastrointestinal Oncology, National Cancer Center Hospital East, Kashiwa, Japan; 3 Department of Gastroenterology, Saitama Cancer Center, Saitama, Japan; 4 Department of Medical Oncology, Kagawa University Hospital, Kagawa, Japan; 5 Kobe City Medical Center General Hospital, Kobe, Japan; 6 Aichi Cancer Center Hospital, Nagoya, Japan; 7 The University of Osaka Hospital, Osaka, Japan; 8 MSD K.K., Tokyo, Japan; 9 Experimental Therapeutics, National Cancer Center Hospital East, Kashiwa, Japan

**Keywords:** Immunotherapy, PD-1 inhibitor, Esophageal neoplasms, Japan

## Abstract

**Background:**

After a median study follow-up of 36.6 months, first-line pembrolizumab plus chemotherapy numerically improved overall survival (OS) and progression-free survival (PFS) versus placebo plus chemotherapy in Japanese participants with advanced esophageal cancer in the phase 3 KEYNOTE-590 study. The 5-year follow-up is presented.

**Methods:**

Participants with previously untreated advanced esophageal cancer were randomly assigned 1:1 to pembrolizumab 200 mg or placebo every 3 weeks up to 35 cycles plus chemotherapy (cisplatin 80 mg/m^2^ and 5-fluorouracil 800 mg/m^2^/day). Primary end points were OS and PFS per RECIST v1.1 by investigator; objective response rate (ORR) and safety were secondary. The data cutoff date was July 10, 2023.

**Results:**

In total, 141 of 749... participants were enrolled in Japan. Median study follow-up was 60.6 months (range, 53.8–69.7). Median OS was 17.7 months (95% CI, 13.9–28.5) with pembrolizumab plus chemotherapy versus 11.7 months (95% CI, 9.5–19.0) with placebo plus chemotherapy (HR, 0.65; 95% CI, 0.45–0.94); 60-month rates were 24.0% and 8.5%. Median PFS was 6.3 months (95% CI, 6.0–8.2) versus 6.0 months (95% CI, 4.2–6.2) (HR, 0.57; 95% CI, 0.39–0.83); 60-month rates were 16.9% and 0%. ORRs were 56.8% (95% CI, 44.7–68.2) and 38.8% (95% CI, 27.1–51.5). The median DOR was 8.3 months for pembrolizumab plus chemotherapy and 6.1 months for placebo plus chemotherapy. No new treatment-related adverse events occurred since the prior analysis.

**Conclusions:**

After a median follow-up of 5 years, pembrolizumab plus chemotherapy continues to provide long-term survival outcomes among Japanese participants with advanced esophageal cancer. No new safety signals were observed.

*Clinical trial registration* ClinicalTrials.gov, NCT03189719.

**Supplementary Information:**

The online version contains supplementary material available at https://doi.org/10.1007/s10388-026-01200-8.

## Introduction

Prior to the inclusion of programmed cell death protein 1 (PD-1) inhibitors in the treatment paradigm, median survival for patients with advanced esophageal cancer treated with cisplatin plus 5-fluorouracil was 6.6–9.5 months [1]. Pembrolizumab, a PD-1 inhibitor, has been shown to prolong survival in a variety of cancers, including esophageal cancer [2, 3]. The randomized, double-blind, phase 3 KEYNOTE-590 study was the first global study designed to evaluate the efficacy and safety of pembrolizumab plus chemotherapy versus placebo plus chemotherapy for the first-line treatment of advanced esophageal cancer [3]. Results from the global population showed that pembrolizumab plus chemotherapy significantly improved overall survival (OS) compared with placebo plus chemotherapy, and had a manageable safety profile. These findings redefined practice guidelines and led to the combination becoming a standard treatment option [1, 3]. To date, this combination is approved for use in patients with locally advanced unresectable or metastatic esophageal cancer in several regions including the United States, European Union, and Japan [2, 4, 5].

Subgroup analyses of Japanese participants in KEYNOTE-590 reported results that were consistent with the global population [6, 7]. In the initial analysis of the Japanese subgroup (median study follow-up, 24.4 months), pembrolizumab plus chemotherapy prolonged OS and progression-free survival (PFS) and improved response rates compared with placebo plus chemotherapy [6]. Longer-term follow-up supported these findings. After a median follow-up of 36.6 months, dual primary end points of OS (HR, 0.70; 95% CI, 0.47–1.03) and PFS (HR, 0.57; 95% CI, 0.39–0.83), and the secondary end point of objective response rate (ORR; 56.8% vs. 38.8%) continued to favor pembrolizumab plus chemotherapy over placebo plus chemotherapy [7].

In the 5-year follow-up, pembrolizumab plus chemotherapy continued to provide a clinically meaningful benefit compared with placebo plus chemotherapy, regardless of histology or programmed cell death ligand 1 (PD-L1) status [8]. Here, we report results from 5-year follow-up of Japanese participants in the KEYNOTE-590 study.

## Methods

### Study design and participants

Detailed methodology for KEYNOTE-590 have been published [3]. Briefly, eligible participants had previously untreated, locally advanced unresectable or metastatic esophageal adenocarcinoma, or esophageal squamous cell carcinoma (ESCC), measurable disease per RECIST version 1.1, and an Eastern Cooperative Oncology Group (ECOG) performance status of 0 or 1. Participants were enrolled regardless of tumor PD-L1 status. Histology classification was based on the American Joint Committee on Cancer version 7 guidelines, which were current at the time of study initiation. This subgroup analysis included participants enrolled in Japan. The study was conducted in accordance with principles of Good Clinical Practice and was approved by the appropriate institutional review boards and regulatory agencies. All participants provided written informed consent.

### Treatment

Participants were randomly assigned 1:1 to pembrolizumab 200 mg or saline placebo intravenously every 3 weeks (Q3W) for up to 35 cycles (~ 2 years) plus cisplatin 80 mg/m^2^ intravenously Q3W for ≤ 6 doses and 5-fluorouracil 800 mg/m^2^/day continuous intravenous infusion on days 1–5 Q3W (or per local standard of care, provided the total dose was 4000 mg/m^2^ per 3-week cycle) for ≤ 35 cycles. Treatment continued until confirmed progressive disease (PD), unacceptable toxicity, or participant or investigator decision to withdraw from the trial. Randomization was stratified by geographic region (Asia vs. non-Asia), histology (adenocarcinoma vs. ESCC), and ECOG performance status (0 vs. 1). Crossover between treatment groups was not allowed.

### Assessments and end points

Tumor imaging was performed every 9 weeks until PD was verified, start of new anticancer treatment, withdrawal of consent, or death, whichever occurred first. Response was assessed per RECIST v1.1 by investigator review. Adverse events (AEs) were monitored throughout study treatment and for 30 days after treatment end (90 days for serious AEs, or 30 days if new anticancer therapy is initiated), and were graded according to the National Cancer Institute Common Terminology Criteria for Adverse Events, version 4.0. AEs were determined to be treatment-related by the investigator. Immune-mediated AEs and infusion reactions were based on a list of preferred terms intended to capture known risks of pembrolizumab and considered regardless of attribution to study treatment by the investigator. Survival status was monitored every 12 weeks until death, withdrawal of consent, or the end of the study, whichever occurred first. PD-L1 expression was assessed in archival or newly collected tumor samples using PD-L1 IHC 22C3 pharmDx (Agilent) and was measured using combined positive score (CPS; defined as the number of PD-L1–staining cells [tumor cells, lymphocytes, macrophages] divided by the total number of viable tumor cells, multiplied by 100).

Dual primary end points were overall survival (OS) in participants with ESCC and PD-L1 CPS≥10, ESCC, PD-L1 CPS≥10, and in the overall population, and progression-free survival per RECIST v1.1 by investigator review in participants with ESCC, PD-L1 CPS≥10, and in the overall population. Secondary end points included ORR and duration of response (DOR) per RECIST v1.1 by investigator review in the overall population and in participants with ESCC and PD-L1 CPS≥10, ESCC, and PD-L1 CPS≥10; and safety and tolerability in the overall population. Post hoc exploratory analyses of OS and PFS in participants with and without liver metastases were performed.

### Statistical analyses

Efficacy was analyzed in the intention-to-treat population, which comprised all participants randomly assigned to treatment. Safety was analyzed in the as-treated population, which comprised all randomly assigned participants who received ≥ 1 dose of study treatment. OS, PFS, and DOR were estimated using the Kaplan–Meier method; between group differences were assessed using a Cox regression model with the Efron method of handling ties with treatment as a covariate. Treatment differences in ORR and 95% CIs were assessed using the stratified Miettinen and Nurminen method. Analysis of the Japanese subgroup was not controlled for multiplicity and no alpha was allocated to the comparisons. Details of sample size calculation for the Japanese subgroup have been published [6].

## Results

### Participants

**Fig. 1:**
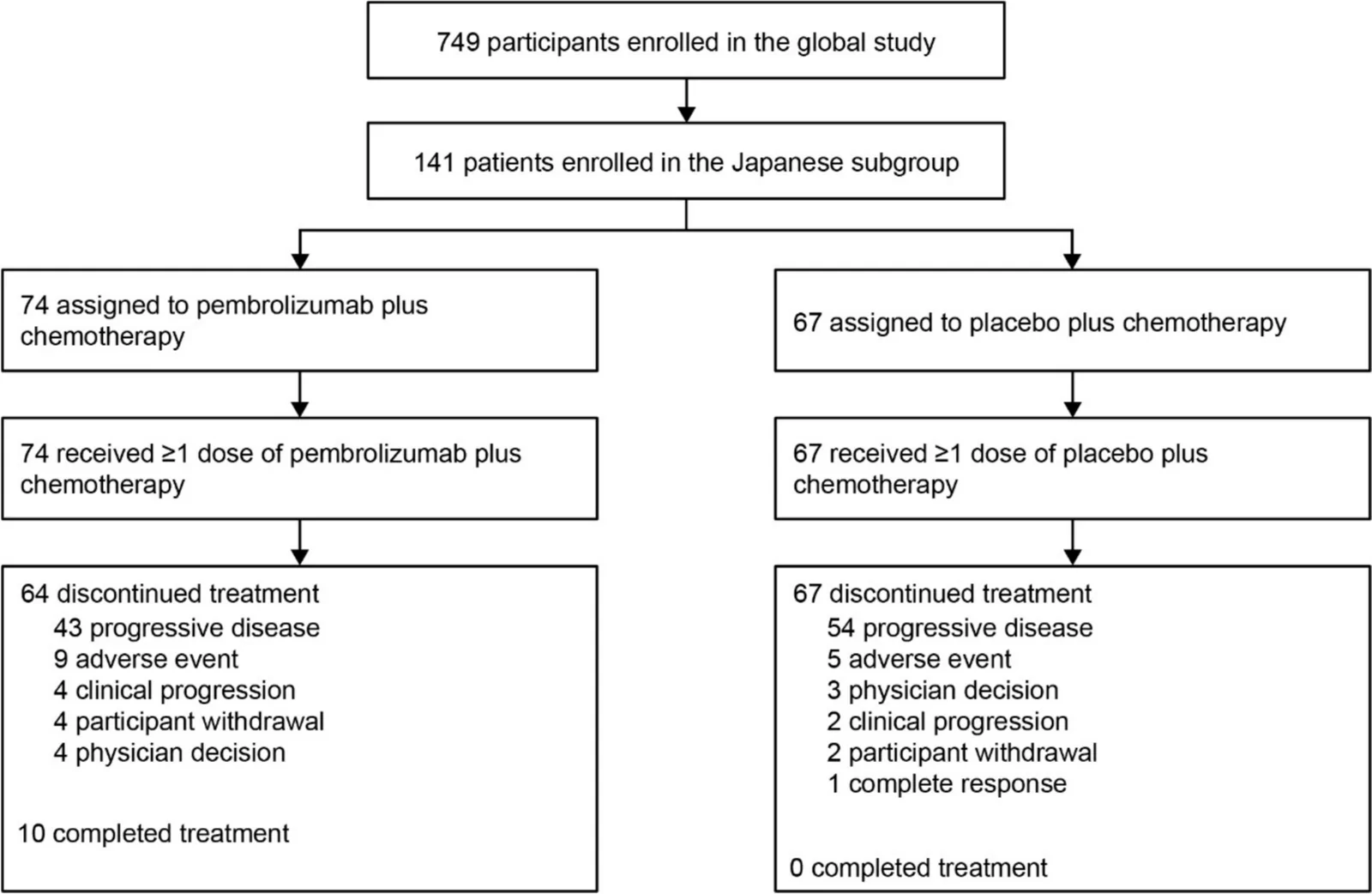
Study profile for participants in the Japanese subgroup

Of 749 participants enrolled in the global KEYNOTE-590 study, 141 were enrolled from Japan (pembrolizumab plus chemotherapy, n=74; placebo plus chemotherapy, n = 67) (Fig. 1). Baseline characteristics were generally balanced between treatment arms (Table 1). The median age of participants was 68 years (range, 32–81); 40 participants (28.4%) had an ECOG performance status of 1, 126 (89.4%) had metastatic disease, 32 (22.7%) had liver metastases, 126 (89.4%) had ESCC, 84 (59.6%) had a PD-L1 CPS≥10, and 76 (53.9%) had ESCC and a PD-L1 CPS ≥ 10. Compared to the intent-to-treat population, Japanese participants were slightly older, had a lower rate of ECOG performance status 1, were ESCC dominant (~ 90%), and had ~ 15% higher CPS>10 population in the pembrolizumab arm.

**Table 1:** Baseline demographics and disease characteristics of participants in the Japanese subgroup

	Pembrolizumab + chemotherapy n = 74	Placebo + chemotherapy n = 67
Age, median (range), years	67.5 (32–81)	68.0 (46–79)
*Age, years*		
< 65	27 (36.5)	25 (37.3)
≥ 65	47 (63.5)	42 (62.7)
*Sex*		
Male	63 (85.1)	61 (91.0)
Female	11 (14.9)	6 (9.0)
*ECOG performance status*		
0	48 (64.9)	53 (79.1)
1	26 (35.1)	14 (20.9)
*Liver metastases*		
Yes	18 (24.3)	14 (20.9)
No	56 (75.7)	53 (79.1)
*Disease status*		
Metastatic	67 (90.5)	59 (88.1)
Unresectable locally advanced	7 (9.5)	8 (11.9)
*Histology*		
ESCC	67 (90.5)	59 (88.1)
Adenocarcinoma	7 (9.5)	8 (11.9)
*PD-L1 CPS*≥*10*	48 (64.9)	36 (53.7)
ESCC	44 (59.5)	32 (47.8)
Adenocarcinoma	4 (5.4)	4 (6.0)
*PD-L1 CPS*<*10*	21 (28.4)	30 (44.8)
ESCC	18 (24.3)	26 (38.8)
Adenocarcinoma	3 (4.1)	4 (6.0)
*PD-L1 not evaluable or missing*	5 (6.8)	1 (1.5)

Data are n (%) unless otherwise stated

CPS, combined positive score; ECOG, Eastern cooperative oncology group; ESCC, esophageal squamous cell carcinoma

Median time from randomization to data cutoff (July 10, 2023) was 60.6 months (range, 53.8–69.7). As of the data cutoff date, 10 participants assigned to pembrolizumab plus chemotherapy had completed treatment and 64 discontinued treatment, primarily because of PD (n = 43 [58.1%]) (Fig. 1). All 67 participants assigned to placebo plus chemotherapy had discontinued treatment, primarily because of PD (n = 54 [80.6%]).

Of participants in the pembrolizumab plus chemotherapy group, 10 (13.5%) underwent conversion surgery and 50 (67.6%) received subsequent systemic therapy, most commonly (≥ 20%) paclitaxel (n = 36 [48.6%]), fluorouracil (n = 23 [31.1%]), and nivolumab (n = 13 [17.6%]). Among all participants in the placebo plus chemotherapy group, 6 (9.0%) underwent conversion surgery and 52 (77.6%) received subsequent systemic therapy, most commonly (≥ 20%) paclitaxel (n = 39 [58.2%]), fluorouracil (n = 17 [25.4%]), and nivolumab (n = 18 [26.9%]). Among participants who underwent conversion surgery in the Japan subgroup, 6 participants (60%) in the pembrolizumab plus chemotherapy group and 4 (67%) in the placebo plus chemotherapy group achieved long-term survival beyond 50 months and were alive at the data cutoff date. Overall, no meaningful difference was observed between the two groups.

### Efficacy

**Fig. 2:**
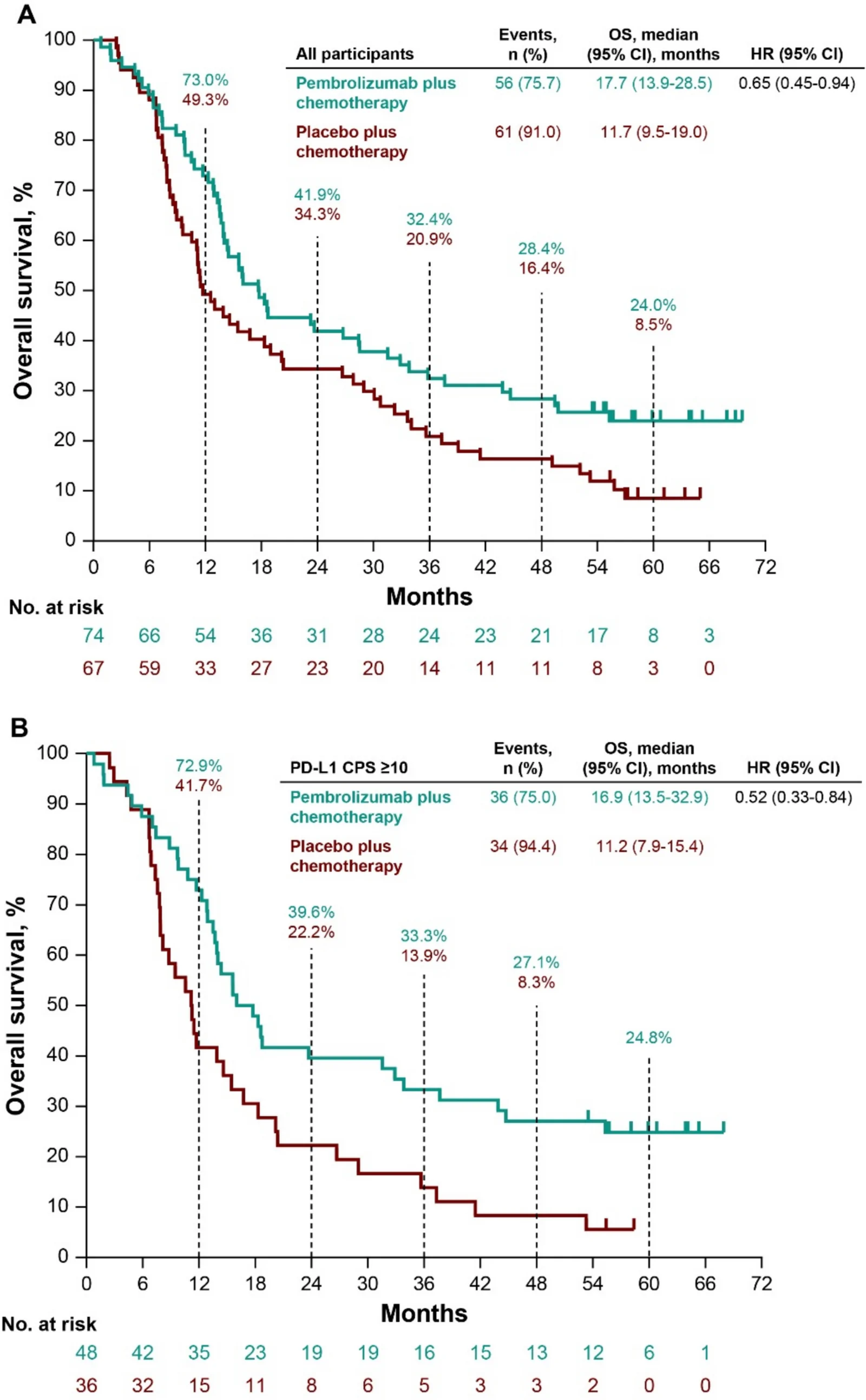

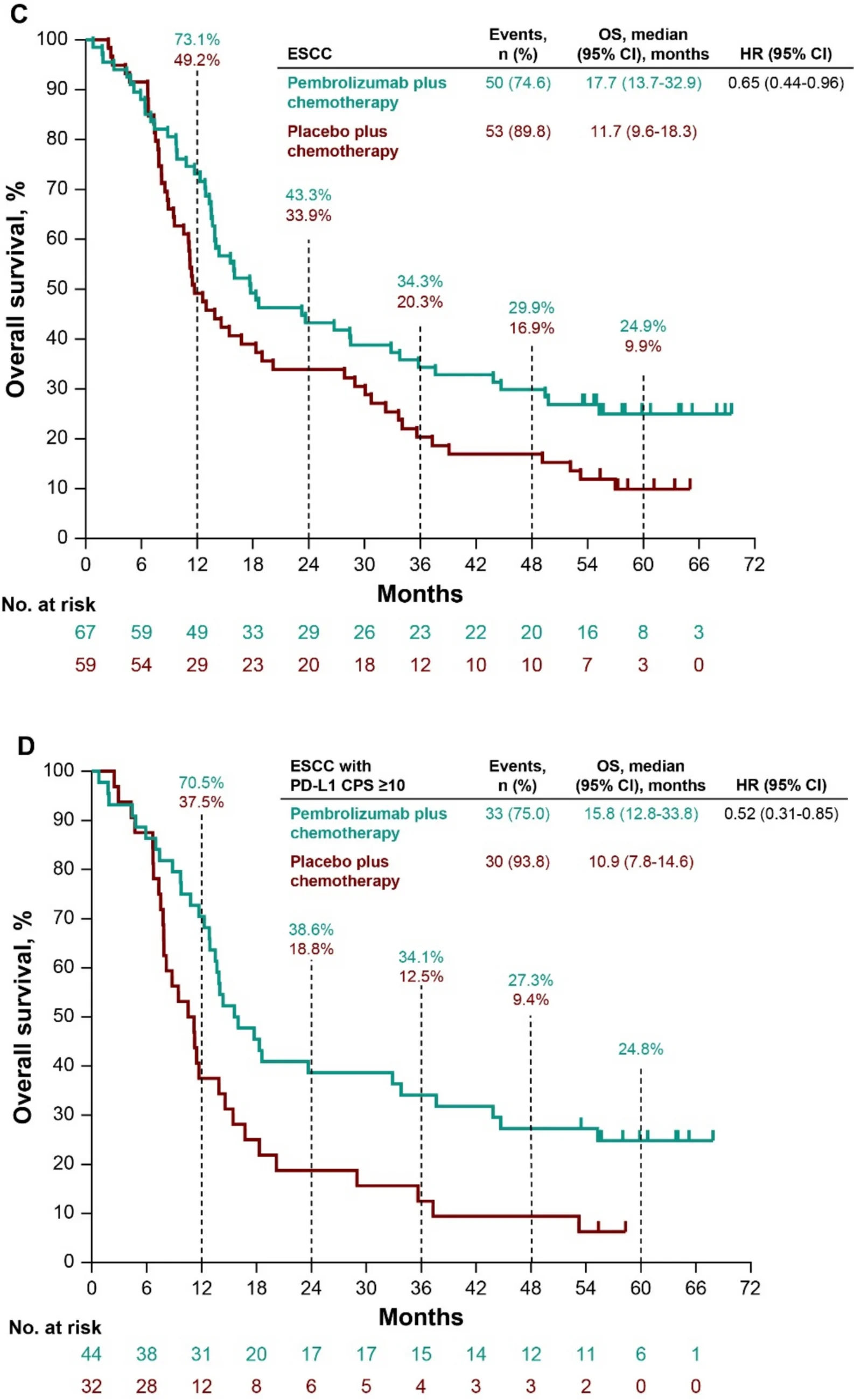

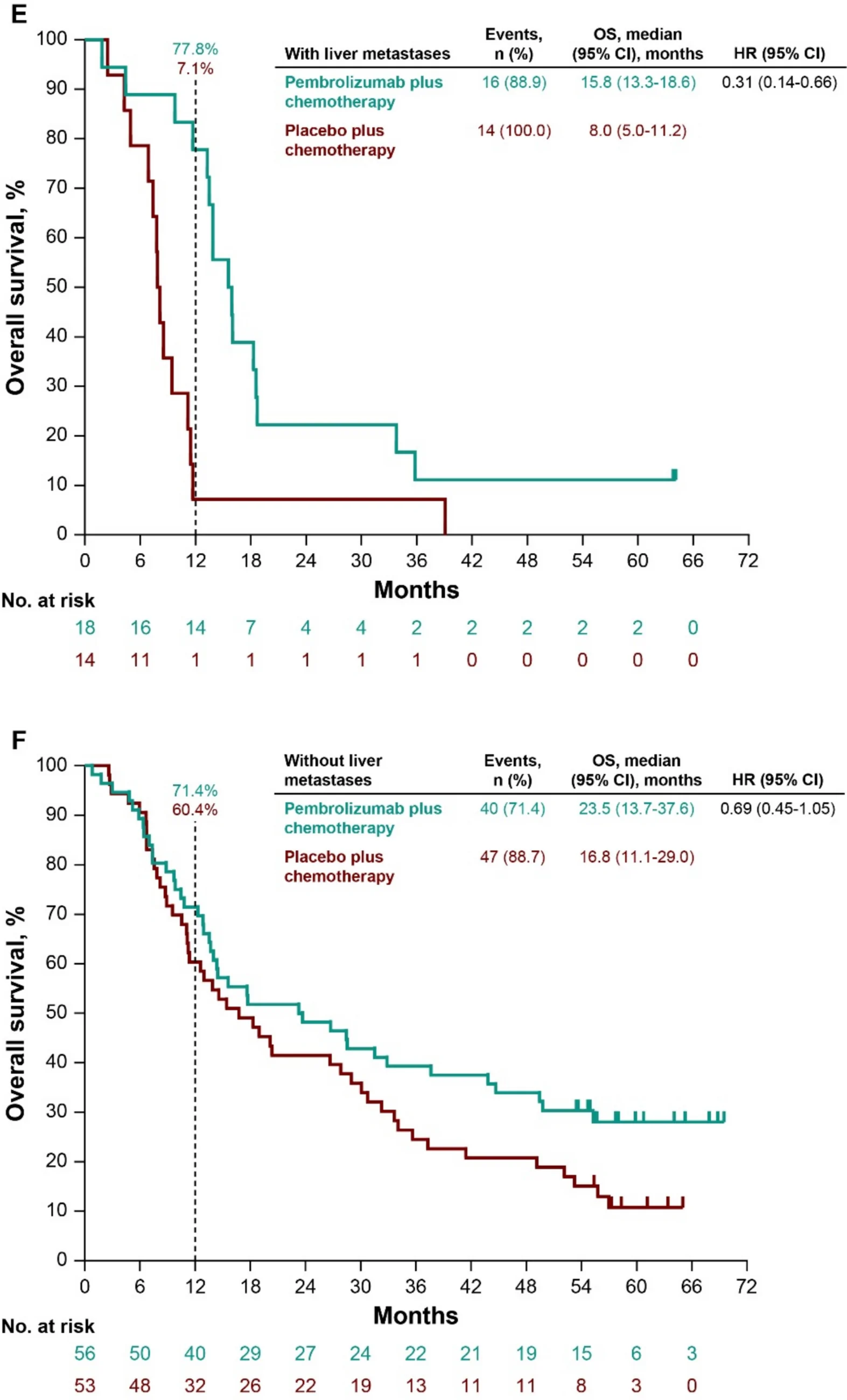
Kaplan–Meier estimates of overall survival in the Japanese subgroup in **A** all participants, **B** participants with PD-L1 CPS≥10, **C** participants with ESCC, **D** participants with ESCC and PD-L1 CPS≥10, **E** participants with liver metastases, and **F** participants without liver metastases

Median OS in the overall Japanese subgroup was 17.7 months (95% CI, 13.9–28.5) for pembrolizumab plus chemotherapy versus 11.7 months (95% CI, 9.5–19.0) for placebo plus chemotherapy (HR, 0.65; 95% CI, 0.45–0.94) (Fig. 2A). The estimated 60-month OS was 24.0% for pembrolizumab plus chemotherapy and 8.5% for placebo plus chemotherapy. Among the prespecified subgroups, median OS was 16.9 months (95% CI, 13.5–32.9) for pembrolizumab plus chemotherapy versus 11.2 months (95% CI, 7.9–15.4) for placebo plus chemotherapy (HR, 0.52; 95% CI, 0.33–0.84) for participants with PD-L1 CPS≥10 (Fig. 2B), 17.7 months (95% CI, 13.7–32.9) versus 11.7 months (95% CI, 9.6–18.3) for participants with ESCC (HR, 0.65; 95% CI, 0.44–0.96) (Fig. 2C), and 15.8 months (95% CI, 12.8–33.8) versus 10.9 months (95% CI, 7.8–14.6) for participants with ESCC and PD-L1 CPS≥10 (HR, 0.52; 95% CI, 0.31–0.85) (Fig. 2D). By liver metastases status, the median OS was 15.8 months (95% CI, 13.3–18.6) for pembrolizumab plus chemotherapy versus 8.0 months (95% CI, 5.0–11.2) for placebo-chemotherapy (HR, 0.31; 95% CI, 0.14–0.66) in participants with liver metastases (Fig. 2E), and 23.5 months (95% CI, 13.7–37.6) versus 16.8 months (95% CI, 11.1–29.0) in participants without liver metastases (HR, 0.69; 95% CI, 0.45–1.05) (Fig. 2F).

**Fig. 3:**
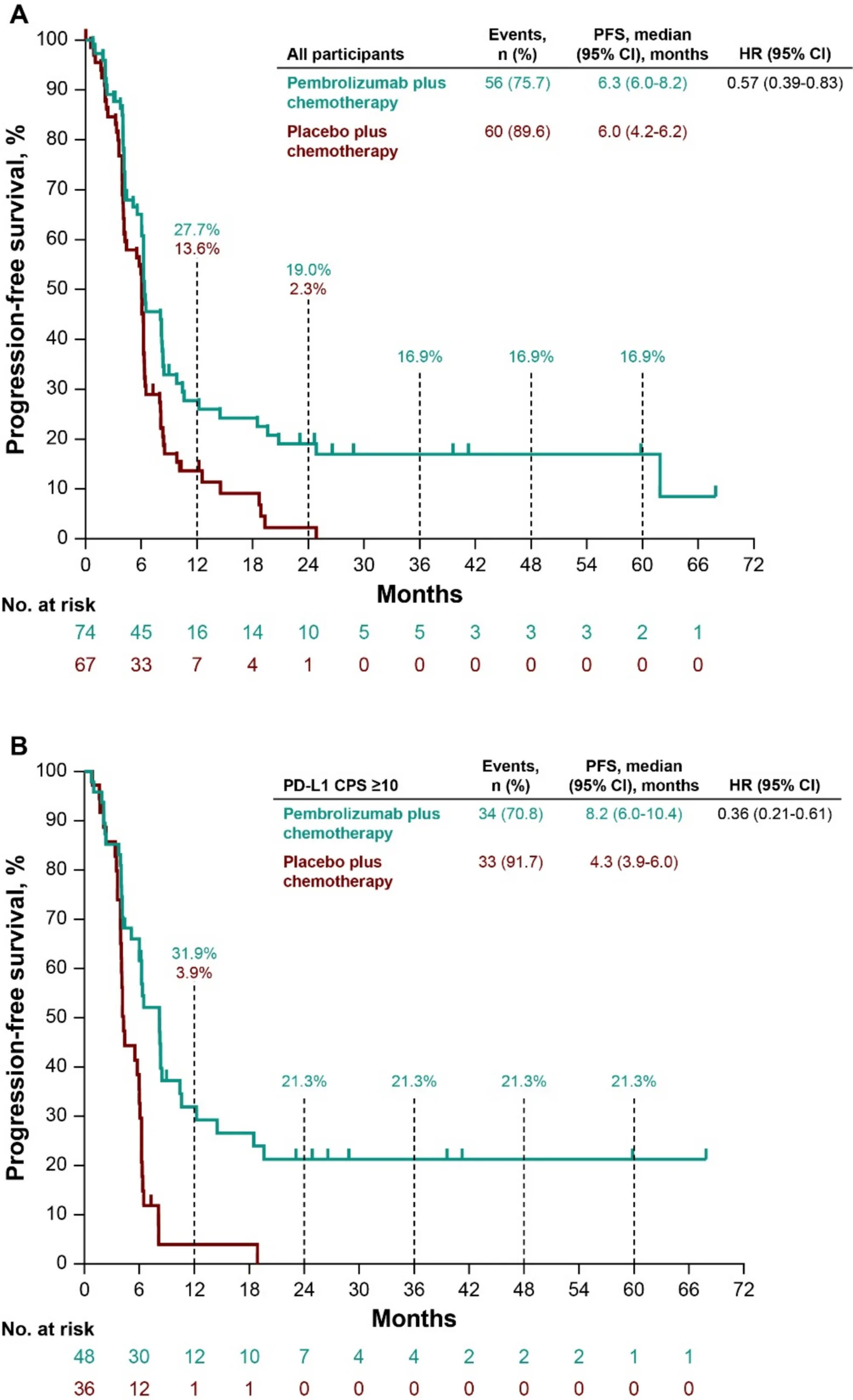

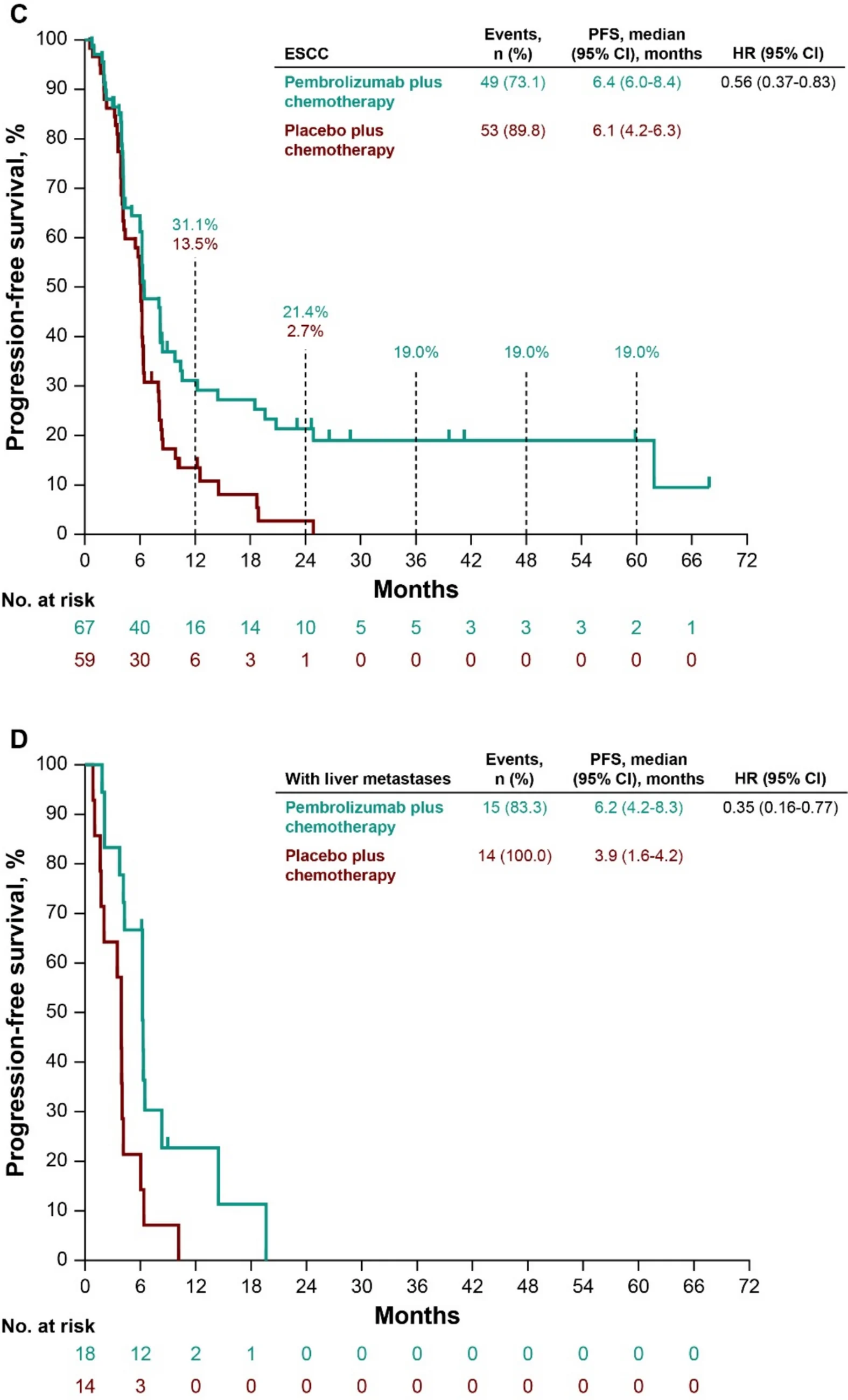

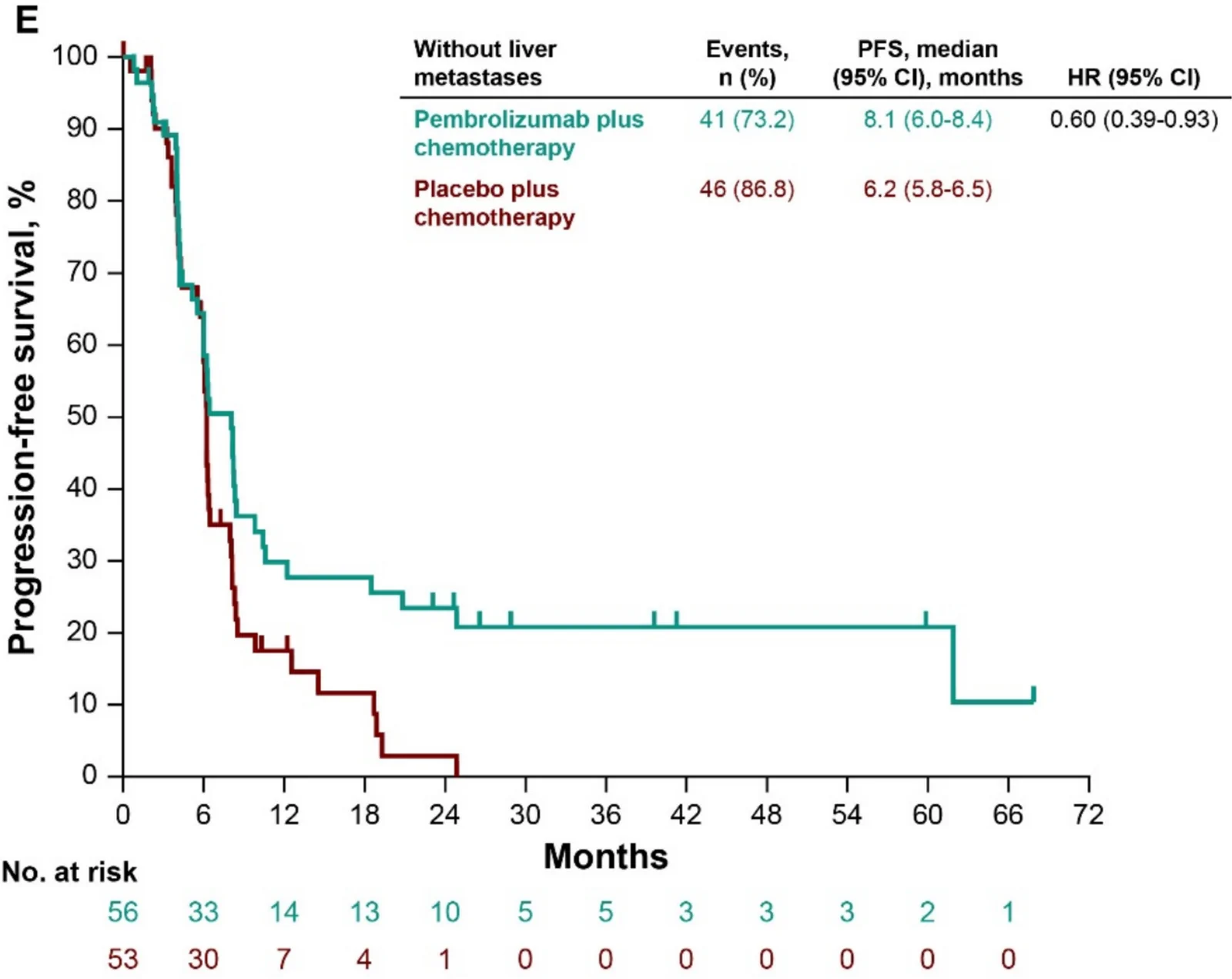
Kaplan–Meier estimates of progression-free survival in the Japanese subgroup in **A** all participants, **B** participants with PD-L1 CPS≥10 **C** participants with ESCC, **D** participants with liver metastases, and **E** participants without liver metastase

Median PFS in the overall Japanese subgroup was 6.3 months (95% CI, 6.0–8.2) for pembrolizumab plus chemotherapy versus 6.0 months (95% CI, 4.2–6.2) for placebo plus chemotherapy (HR, 0.57; 95% CI, 0.39–0.83) (Fig. 3A). The estimated 60-month PFS was 16.9% for pembrolizumab plus chemotherapy and 0% for placebo plus chemotherapy. Among the prespecified subgroups, median PFS was 8.2 months (95% CI, 6.0–10.4) for pembrolizumab plus chemotherapy versus 4.3 months (95% CI, 3.9–6.0) for placebo plus chemotherapy (HR, 0.36; 95% CI, 0.21–0.61) for participants with PD-L1 CPS≥10 (Fig. 3B) and 6.4 months (95% CI, 6.0–8.4) versus 6.1 months (95% CI, 4.2–6.3) for participants with ESCC (HR, 0.56; 95% CI, 0.37–0.83) (Fig. 3C). By liver metastasis status, the median PFS was 6.2 months (95% CI, 4.2–8.3) for pembrolizumab plus chemotherapy versus 3.9 months (95% CI, 1.6–4.2) for placebo-chemotherapy (HR, 0.35; 95% CI, 0.16–0.77) in participants with liver metastases (Fig. 3D), and 8.1 months (95% CI, 6.0–8.4) versus 6.2 months (95% CI, 5.8–6.5) in participants without liver metastases (HR, 0.60; 95% CI, 0.39–0.93) (Fig. 3E).

In the overall Japanese subgroup, the ORR was 56.8% (95% CI, 44.7–68.2; 4 complete responses [CR], 38 partial responses [PR]) for pembrolizumab plus chemotherapy and 38.8% (95% CI, 27.1–51.5; 3 CR, 23 PR) for placebo plus chemotherapy (Table 2). The median DOR was 8.3 months (range, 1.2 + to 65.9 +) for pembrolizumab plus chemotherapy and 6.1 months (range, 3.5 to 22.8) for placebo plus chemotherapy. An estimated 26.7% of participants in the pembrolizumab plus chemotherapy group remained in response at 54 months (Table 2).

**Table 2:** Summary of best objective response for participants in the Japanese subgroup

	Pembrolizumab + chemotherapy n = 74	Placebo + chemotherapy n = 67
*ORR,*^*a*^* % (95% CI)*	56.8 (44.7–68.2)	38.8 (27.1–51.5)
Difference, % (95% CI)	18.0 (1.4–33.5)	
*BOR, n (%)*		
CR	4 (5.4)	3 (4.5)
PR	38 (51.4)	23 (34.3)
SD	25 (33.8)	30 (44.8)
PD	5 (6.8)	10 (14.9)
Not evaluable^b^	2 (2.7)	1 (1.5)
**Time to response,**^**a**^** median (range), months**	2.0 (1.9 to 8.3)	2.0 (1.9 to 4.4)
**DOR,**^**c**^** median (range), months**	8.3 (1.2 + to 65.9 +)	6.1 (3.5 to 22.8)
*Participants with extended DOR,*^*a,c*^* %*		
≥ 54 months	26.7	NR

CR, complete response; DOR, duration of response; NR, not reached; ORR, objective response rate; PD, progressive disease; PR, partial response; RECIST v1.1, Response Evaluation Criteria in Solid Tumors, version 1.1; SD, stable disease

+ indicates there was no PD by the time of last disease assessment

^a^Includes participants who experienced CR and PR

^b^Postbaseline assessment available but not evaluable or response of CR, PR, or SD < 6 weeks from randomization

^c^From product-limit (Kaplan–Meier) method for censored data

In the prespecified subgroups, the ORR was 60.4% (95% CI, 45.3–74.2; 3 CR, 26 PR) for pembrolizumab plus chemotherapy and 30.6% (95% CI, 16.3–48.1; 1 CR, 10 PR) for placebo plus chemotherapy among participants with PD-L1 CPS≥10, 56.7% (95% CI, 44.0–68.8; 4 CR, 34 PR) and 40.7% (95% CI, 28.1–54.3; 2 CR, 22 PR) among participants with ESCC, and 59.1% (95% CI, 43.2–73.7; 3 CR, 23 PR) and 31.3% (95% CI, 16.1–50.0; 1 CR, 9 PR) among participants with ESCC and PD-L1 CPS≥10 (Supplementary Table 1). The median DOR was 10.4 months (range, 2.3 + to 65.9 +) for pembrolizumab plus chemotherapy and 4.4 months (range, 3.5–17.0) for placebo plus chemotherapy among participants with PD-L1 CPS≥10, 10.4 months (range, 1.2 + to 65.9 +) and 6.1 months (range, 3.5–22.8) among participants with ESCC, and 10.5 months (range, 2.3 + to 65.9 +) and 4.4 months (range, 3.5–17.0) among participants with ESCC and PD-L1 CPS≥10 (Supplementary Table 1).

### Safety

All-cause AEs of any grade occurred in all participants in the pembrolizumab plus chemotherapy and placebo plus chemotherapy groups (Supplementary Table 2). Treatment-related AEs occurred in 73 participants (98.6%) in the pembrolizumab plus chemotherapy group and 66 (98.5%) in the placebo plus chemotherapy group (Table 3); grade 3–4 events occurred in 53 (71.6%) and 40 (59.7%) participants, respectively. Three participants died because of treatment-related AEs: 2 (2.7%) in the pembrolizumab plus chemotherapy group (interstitial lung disease, pneumonitis) and 1 (1.5%) in the placebo plus chemotherapy group (interstitial lung disease). No new treatment-related deaths occurred since the prior analysis [7]. At data cutoff, among participants who experienced an OS event, the primary cause of death was malignant neoplasm progression in 50 of 56 participants (89%) in the pembrolizumab plus chemotherapy group and 59 of 61 participants (97%) in the placebo plus chemotherapy group. Non-cancer causes of death were acute interstitial lung disease/pneumonitis (4%), acute myocardial infarction (2%), aspiration pneumonia (2%), respiratory failure (2%), and unknown causes (2%) in the pembrolizumab plus chemotherapy group versus asthenia (2%) and interstitial lung disease/pneumonitis (2%) in the placebo plus chemotherapy group. Immune-mediated AEs and infusion reactions occurred in 25 participants (33.8%) in the pembrolizumab plus chemotherapy group and 18 (26.9%) in the placebo plus chemotherapy group; grade 3–4 events occurred in 8 (10.8%) and 0 participants in each treatment group respectively (Table 3).

**Table 3:** Treatment-related adverse events and immune-mediated adverse event and infusion reactions in the Japanese subgroup

	Pembrolizumab + chemotherapy, n = 74			Placebo + chemotherapy n = 67		
	Any grade	Grade 3–4	Grade 5	Any grade	Grade 3–4	Grade 5
*Treatment-related AEs*^*a*^	**73 (98.6)**	**53 (71.6)**	**2 (2.7)**	**66 (98.5)**	**40 (59.7)**	**1 (1.5)**
Decreased appetite	58 (78.4)	4 (5.4)	0	39 (58.2)	7 (10.4)	0
Nausea	55 (74.3)	2 (2.7)	0	42 (62.7)	3 (4.5)	0
Neutrophil count decreased	45 (60.8)	29 (39.2)	0	38 (56.7)	22 (32.8)	0
Stomatitis	42 (56.8)	3 (4.1)	0	35 (52.2)	2 (3.0)	0
White blood cell count decreased	35 (47.3)	14 (18.9)	0	22 (32.8)	6 (9.0)	0
Anemia	28 (37.8)	11 (14.9)	0	29 (43.3)	11 (16.4)	0
Fatigue	26 (35.1)	2 (2.7)	0	10 (14.9)	1 (1.5)	0
Malaise	26 (35.1)	1 (1.4)	0	23 (34.3)	2 (3.0)	0
Constipation	23 (31.1)	0	0	19 (28.4)	0	0
Hiccups	23 (31.1)	0	0	15 (22.4)	0	0
Blood creatinine increased	20 (27.0)	0	0	22 (32.8)	0	0
Diarrhea	20 (27.0)	3 (4.1)	0	18 (26.9)	2 (3.0)	0
Alopecia	18 (24.3)	0	0	13 (19.4)	0	0
Platelet count decreased	17 (23.0)	1 (1.4)	0	14 (20.9)	1 (1.5)	0
Dysgeusia	16 (21.6)	0	0	13 (19.4)	0	0
Peripheral sensory neuropathy	15 (20.3)	1 (1.4)	0	14 (20.9)	0	0
Lymphocyte count decreased	12 (16.2)	6 (8.1)	0	8 (11.9)	2 (3.0)	0
Edema	11 (14.9)	0	0	8 (11.9)	0	0
Hyponatremia	10 (13.5)	9 (12.2)	0	16 (23.9)	8 (11.9)	0
Vomiting	8 (10.8)	1 (1.4)	0	10 (14.9)	0	0
Vasculitis	6 (8.1)	0	0	7 (10.4)	0	0
Rash	5 (6.8)	0	0	8 (11.9)	0	0
Hyperkalemia	1 (1.4)	1 (1.4)	0	7 (10.4)	2 (3.0)	0
*Immune-mediated AEs and infusion reactions*	**25 (33.8)**	**8 (10.8)**	**2 (2.7)**	**18 (26.9)**	**0**	**1 (1.5)**
Adrenal insufficiency	1 (1.4)	1 (1.4)	0	2 (3.0)	0	0
Colitis	4 (5.4)	2 (2.7)	0	1 (1.5)	0	0
Gastritis	0	0	0	1 (1.5)	0	0
Hepatitis	1 (1.4)	1 (1.4)	0	0	0	0
Hyperthyroidism	3 (4.1)	0	0	1 (1.5)	0	0
Hypophysitis	2 (2.7)	0	0	0	0	0
Hypothyroidism	7 (9.5)	0	0	5 (7.5)	0	0
Infusion reactions	2 (2.7)	0	0	1 (1.5)	0	0
Nephritis	0	0	0	1 (1.5)	0	0
Pneumonitis	5 (6.8)	1 (1.4)	2 (2.7)	1 (1.5)	0	1 (1.5)
Severe skin reactions	4 (5.4)	4 (5.4)	0	0	0	0
Type 1 diabetes mellitus	1 (1.4)	1 (1.4)	0	0	0	0
Vasculitis	6 (8.1)	0	0	8 (11.9)	0	0

## Discussion

In this 5-year follow-up of Japanese participants with advanced esophageal cancer enrolled in KEYNOTE-590, pembrolizumab plus chemotherapy is associated with numerically improved OS and PFS regardless of histology or PD-L1 status. The 60-month OS and PFS estimates of 24.0% and 16.9%, respectively, were observed in the Japanese subgroup. While the overall efficacy outcomes in this 5-year analysis are directionally consistent with prior reports, the extended follow-up provides important new insights into the long-term survival patterns of patients from Japan treated with pembrolizumab plus chemotherapy. With longer observation, OS and PFS curves showed sustained separation between treatment groups through 5 years, with the persistence of a long-term survival tail in the pembrolizumab plus chemotherapy group. Although a distinct plateau was not clearly observed, approximately one-fourth of participants remained alive at 60 months, and long-term PFS was only observed in the pembrolizumab plus chemotherapy group. The continued separation without late convergence and the presence of long-term survivors were not fully characterized at the earlier data cutoff, highlighting the key value of extended follow-up.

No additional responses were observed in the overall Japanese subgroup since the prior analysis [7]. Median duration of response remained at 8.3 months in the pembrolizumab plus chemotherapy group and 6.1 months in the placebo plus chemotherapy group [7]; an estimated 26.7% of participants in the pembrolizumab plus chemotherapy group and 0% in the placebo plus chemotherapy group remained in response at 54 months. These findings indicate that long-term survival benefit is driven by durable early responses rather than late-onset activity. The safety profile continued to be manageable and consistent with prior reports, both in the Japanese subgroup and the global population [3, 6, 7]. Pembrolizumab plus chemotherapy may provide a small opportunity (+ 5%) for conversion surgery compared to placebo chemotherapy; however, the rate of subsequent systemic therapy with placebo plus chemotherapy was 10% higher than with pembrolizumab plus chemotherapy. Although the potential impact of subsequent therapy and post-treatment surgery on survival is uncertain, these results may serve as a baseline for future studies.

Efficacy results observed in the current analysis were similar to those observed in the 5-year follow-up of the global population [8]. OS in the global population was prolonged with pembrolizumab plus chemotherapy versus placebo plus chemotherapy (HR, 0.72; 95% CI, 0.62–0.84), and the 5-year OS rates were 10.6% and 3.0%, respectively [8]. Median PFS in the global population was also prolonged with pembrolizumab plus chemotherapy (HR, 0.64; 95% CI, 0.54–0.75); 5-year PFS rates were 5.5% and 0%, respectively. The ORR in the global population was higher in the pembrolizumab plus chemotherapy group versus the placebo plus chemotherapy group (45.0% vs. 29.3%). Results from subgroup analyses of participants with PD-L1 CPS≥10, ESCC, ESCC with PD-L1 CPS≥10, and with and without liver metastasis also consistently showed a benefit with pembrolizumab plus chemotherapy versus placebo plus chemotherapy. Unlike some malignancies in which immune checkpoint inhibitors produce a pronounced survival curve plateau, esophageal cancer is associated with substantial mortality related to both disease progression and non-cancer comorbidities. At data cutoff, the overwhelming majority of deaths in both treatment groups were attributable to malignant neoplasm progression rather than competing comorbidities. The absence of a clearly flat OS plateau in this analysis, therefore, likely reflects ongoing late events rather than substantial mortality from non-cancer causes or a loss of durable treatment benefit. Notably, long-term survival beyond 5 years was observed at a markedly higher frequency with pembrolizumab plus chemotherapy compared with placebo plus chemotherapy, supporting the presence of sustained clinical benefit in a subset of patients. Several larger trials such as CheckMate 648 and RATIONALE-306 have also failed to show a plateau with immune checkpoint inhibitors plus chemotherapy; therefore, this finding likely reflects the real-world outcomes of chemoimmunotherapy in esophageal cancer.

Limited data are available regarding long-term benefit of PD-1 inhibitors as first-line treatment for esophageal cancer, particularly for patients of Japanese descent. Three year follow-up of the phase 3 ATTRACTION study conducted in Asian patients (55% from Japan), confirmed a long-term benefit with nivolumab-chemotherapy versus placebo plus chemotherapy in patients with previously untreated advanced gastric cancer [9]. No difference was observed in OS; median OS was 17.45 months in the nivolumab plus chemotherapy group versus 17.15 months in the placebo plus chemotherapy group (HR, 0.89; 95% CI, 0.75–1.05); 3-year OS, 23.9% versus 19.4%. PFS was longer with nivolumab plus chemotherapy compared with placebo plus chemotherapy; median PFS was 10.94 months versus 8.48 months (HR, 0.67; 95% CI, 0.55–0.82); 3-year PFS, 27.4% versus 12.3%. However, a large proportion of patients received post-study therapy, which may have likely contributed to the minimal difference observed in OS between treatment arms. No specific subgroup analyses of Japanese patients were reported. Four year follow-up of the global phase 3 CheckMate 649 study showed that first-line nivolumab plus chemotherapy had long-term efficacy and manageable safety compared with chemotherapy alone in patients with advanced gastric cancer or gastroesophageal junction cancer or esophageal adenocarcinoma; no analyses of Japanese patients were reported [10]. Similarly, a 61-month follow-up of the global CheckMate 648 study reported a clinically meaningful OS benefit with first-line nivolumab plus chemotherapy compared with chemotherapy alone in patients with advanced ESCC (HR, 0.77; 95% CI, 0.65–0.92) [11]. At a 45.1 month follow-up of CheckMate 648, patients from Japan also reported a clinically meaningful OS benefit with nivolumab plus chemotherapy versus chemotherapy alone (HR, 0.81) [12]. Although cross-trial comparisons should be interpreted with caution, the long-term outcomes observed in the current Japanese subgroup analysis of KEYNOTE-590 are broadly consistent with emerging long-term data from CheckMate 648.

This analysis is limited by its post hoc nature and the relatively low number of participants enrolled in Japan. Within the limitations of this post hoc subgroup analysis, exploratory observations suggest that patients with PD-L1 CPS≥10 and ESCC histology may more likely experience long-term benefit, consistent with earlier analyses. In addition, a subset of patients who underwent conversion surgery achieved long-term survival in both treatment groups, underscoring the potential contribution of multimodality treatment in selected patients. However, to the authors’ knowledge, this analysis is the longest follow-up of a first-line immune checkpoint inhibitor in Japanese patients, and therefore, provides valuable data for guiding clinician decisions in Japan.

Results of this 5-year analysis support the continued use of pembrolizumab plus chemotherapy as a standard of care first-line treatment option in Japanese patients with advanced esophageal cancer and highlight the potential long-term benefits of PD-1 inhibitors in these patients.

## Supplementary Information

Below is the link to the electronic supplementary material.

Supplementary file1 (DOCX 23 KB)

## Data Availability

Merck Sharp & Dohme LLC, a subsidiary of Merck & Co., Inc., Rahway, NJ, USA (MSD) is committed to providing qualified scientific researchers access to anonymized data and clinical study reports from the company’s clinical trials for the purpose of conducting legitimate scientific research. MSD is also obligated to protect the rights and privacy of trial participants and, as such, has a procedure in place for evaluating and fulfilling requests for sharing company clinical trial data with qualified external scientific researchers. The MSD data sharing website (available at: https://externaldatasharing-msd.com/) outlines the process and requirements for submitting a data request. Applications will be promptly assessed for completeness and policy compliance. Feasible requests will be reviewed by a committee of MSD subject matter experts to assess the scientific validity of the request and the qualifications of the requestors. In line with data privacy legislation, submitters of approved requests must enter into a standard data-sharing agreement with MSD before data access is granted. Data will be made available for request after product approval in the US and EU or after product development is discontinued. There are circumstances that may prevent MSD from sharing requested data, including country or region-specific regulations. If the request is declined, it will be communicated to the investigator. Access to genetic or exploratory biomarker data requires a detailed, hypothesis-driven statistical analysis plan that is collaboratively developed by the requestor and MSD subject matter experts; after approval of the statistical analysis plan and execution of a data-sharing agreement, MSD will either perform the proposed analyses and share the results with the requestor or will construct biomarker covariates and add them to a file with clinical data that is uploaded to an analysis portal so that the requestor can perform the proposed analyses.
