# Correction: First-line pembrolizumab plus chemotherapy versus chemotherapy alone for advanced esophageal cancer: 5-year extended follow-up in the Japanese subgroup of KEYNOTE-590

**DOI:** 10.1007/s10388-026-01249-5

**Published:** 2026-10-04

**Authors:** Ken Kato, Takashi Kojima, Hiroki Hara, Akihito Tsuji, Hisateru Yasui, Kei Muro, Taroh Satoh, Naoyoshi Yatsuzuka, Tomoko Sakata, Shirong Han, Toshihiko Doi

**Affiliations:** 1 Department of Head and Neck, Esophageal Medical Oncology, National Cancer Center Hospital, 5-1-1 Tsukiji, Chuo-Ku, Tokyo, 104-0045 Japan; 2 Department of Gastroenterology and Gastrointestinal Oncology, National Cancer Center Hospital East, Kashiwa, Japan; 3 Department of Gastroenterology, Saitama Cancer Center, Saitama, Japan; 4 Department of Medical Oncology, Kagawa University Hospital, Kagawa, Japan; 5 Kobe City Medical Center General Hospital, Kobe, Japan; 6 Aichi Cancer Center Hospital, Nagoya, Japan; 7 The University of Osaka Hospital, Osaka, Japan; 8 MSD K.K., Tokyo, Japan; 9 Experimental Therapeutics, National Cancer Center Hospital East, Kashiwa, Japan

**Correction: Esophagus (2026) 23:486–500**

**https://doi.org/10.1007/s10388-026-01200-8**

Following publication of the original article, the authors identified few errors and are corrected in this publication.

1. In the first sentence of Results of the “Abstract” section, the sentence, “In total, 141 of 794… participants were enrolled in Japan. Median study follow-up was 60.6 months (range, 53.8–69.7).” should have been “In total, 141 of 749… participants were enrolled in Japan. Median study follow-up was 60.6 months (range, 53.8–69.7).”
2. In Table 3, several values describing treatment-related adverse events and immune-mediated adverse events/infusion reactions in the Japanese subgroup were incorrectly reported. For completeness and transparency, the old incorrect and correct versions are displayed below.

**Incorrect Version:**

**Figure:**
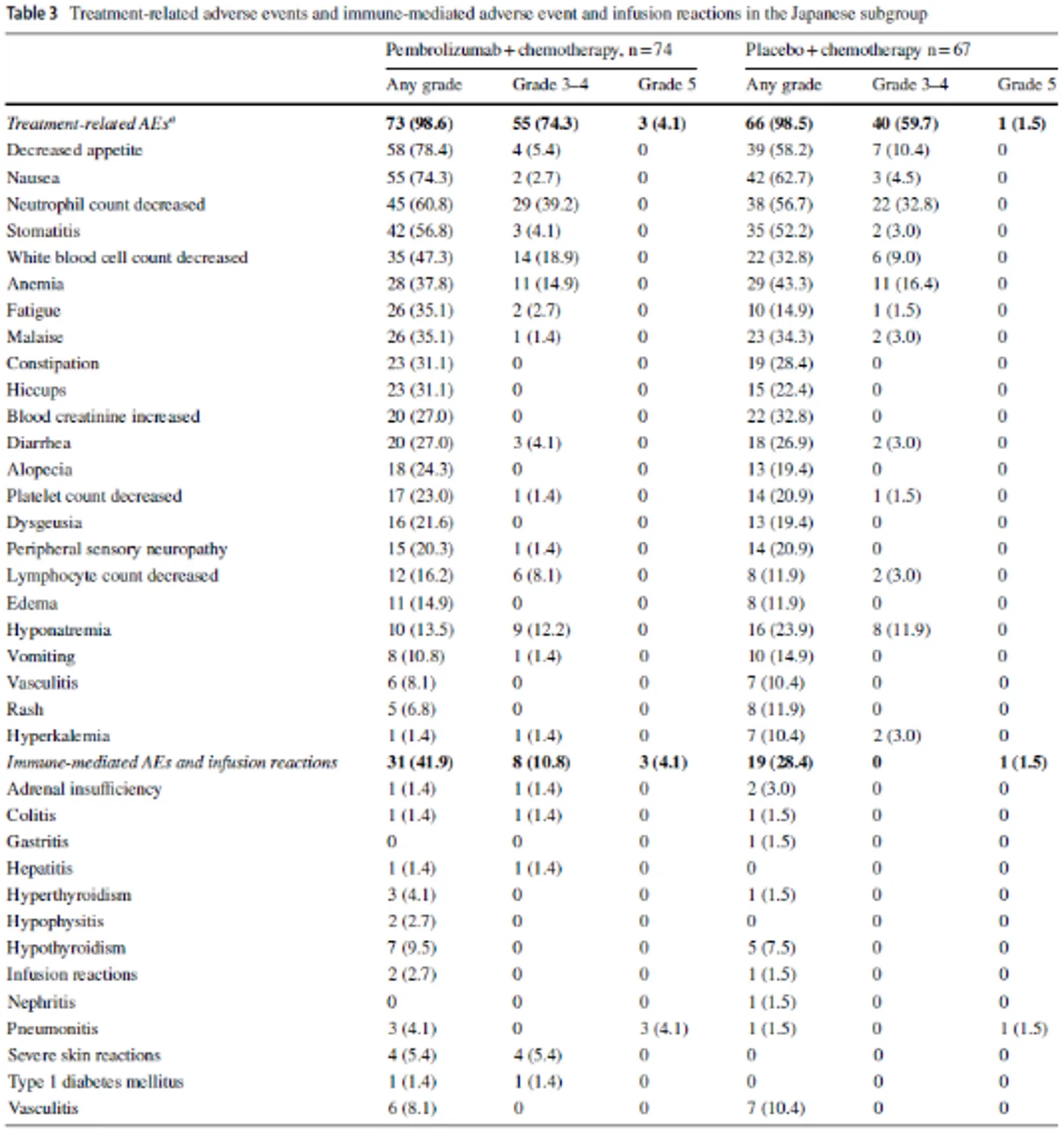

**Correct Version:**

**Table 3:** Treatment-related adverse events and immune-mediated adverse events and infusion reactions in the Japanese subgroup

	Pembrolizumab + chemotherapy (*n* = 74)			Placebo + chemotherapy (*n* = 67)		
	Any grade	Grade 3–4	Grade 5	Any grade	Grade 3–4	Grade 5
**Treatment-related AEs** ^**a**^	**73 (98.6)**	**53 (71.6)**	**2 (2.7)**	**66 (98.5)**	**40 (59.7)**	**1 (1.5)**
Decreased appetite	58 (78.4)	4 (5.4)	0	39 (58.2)	7 (10.4)	0
Nausea	55 (74.3)	2 (2.7)	0	42 (62.7)	3 (4.5)	0
Neutrophil count decreased	45 (60.8)	29 (39.2)	0	38 (56.7)	22 (32.8)	0
Stomatitis	42 (56.8)	3 (4.1)	0	35 (52.2)	2 (3.0)	0
White blood cell count decreased	35 (47.3)	14 (18.9)	0	22 (32.8)	6 (9.0)	0
Anemia	28 (37.8)	11 (14.9)	0	29 (43.3)	11 (16.4)	0
Fatigue	26 (35.1)	2 (2.7)	0	10 (14.9)	1 (1.5)	0
Malaise	26 (35.1)	1 (1.4)	0	23 (34.3)	2 (3.0)	0
Constipation	23 (31.1)	0	0	19 (28.4)	0	0
Hiccups	23 (31.1)	0	0	15 (22.4)	0	0
Blood creatinine increased	20 (27.0)	0	0	22 (32.8)	0	0
Diarrhea	20 (27.0)	3 (4.1)	0	18 (26.9)	2 (3.0)	0
Alopecia	18 (24.3)	0	0	13 (19.4)	0	0
Platelet count decreased	17 (23.0)	1 (1.4)	0	14 (20.9)	1 (1.5)	0
Dysgeusia	16 (21.6)	0	0	13 (19.4)	0	0
Peripheral sensory neuropathy	15 (20.3)	1 (1.4)	0	14 (20.9)	0	0
Lymphocyte count decreased	12 (16.2)	6 (8.1)	0	8 (11.9)	2 (3.0)	0
Edema	11 (14.9)	0	0	8 (11.9)	0	0
Hyponatremia	10 (13.5)	9 (12.2)	0	16 (23.9)	8 (11.9)	0
Vomiting	8 (10.8)	1 (1.4)	0	10 (14.9)	0	0
Vasculitis	6 (8.1)	0	0	7 (10.4)	0	0
Rash	5 (6.8)	0	0	8 (11.9)	0	0
Hyperkalemia	1 (1.4)	1 (1.4)	0	7 (10.4)	2 (3.0)	0
**Immune-mediated AEs and infusion reactions**	**25 (33.8)**	**8 (10.8)**	**2 (2.7)**	**18 (26.9)**	**0**	**1 (1.5)**
Adrenal insufficiency	1 (1.4)	1 (1.4)	0	2 (3.0)	0	0
Colitis	4 (5.4)	2 (2.7)	0	1 (1.5)	0	0
Gastritis	0	0	0	1 (1.5)	0	0
Hepatitis	1 (1.4)	1 (1.4)	0	0	0	0
Hyperthyroidism	3 (4.1)	0	0	1 (1.5)	0	0
Hypophysitis	2 (2.7)	0	0	0	0	0
Hypothyroidism	7 (9.5)	0	0	5 (7.5)	0	0
Infusion reactions	2 (2.7)	0	0	1 (1.5)	0	0
Nephritis	0	0	0	1 (1.5)	0	0
Pneumonitis	5 (6.8)	1 (1.4)	2 (2.7)	1 (1.5)	0	1 (1.5)
Severe skin reactions	4 (5.4)	4 (5.4)	0	0	0	0
Type 1 diabetes mellitus	1 (1.4)	1 (1.4)	0	0	0	0
Vasculitis	6 (8.1)	0	0	8 (11.9)	0	0

Data are n (%)

*AEs* adverse events

^a^Treatment-related adverse events with incidence of 10% or higher in any treatment group

3. In the subsection “Safety” of the “Results” section, the sentence, “Treatment related AEs occurred in 73 participants (98.6%) in the pembrolizumab plus chemotherapy group and 66 (98.5%) in the placebo plus chemotherapy group (Table 3); grade 3–4 events occurred in 55 (74.3%) and 40 (59.7%) participants, respectively.” should read “Treatment related AEs occurred in 73 participants (98.6%) in the pembrolizumab plus chemotherapy group and 66 (98.5%) in the placebo plus chemotherapy group (Table 3); grade 3–4 events occurred in 53 (71.6%) and 40 (59.7%) participants, respectively.”

The corrections do not affect the interpretation of the study results, discussion, or conclusions of the article. The original article has been corrected.

