# Supplementary material for: First-line pembrolizumab plus chemotherapy versus chemotherapy alone for advanced esophageal cancer: 5-year extended follow-up in the Japanese subgroup of KEYNOTE-590
Source: Esophagus. 2026 Apr 28;23(3):486–500. doi: 10.1007/s10388-026-01200-8 (PMC13319894; doi:10.1007/s10388-026-01200-8)
Supplement: Supplementary file 1 — Supplementary file1 (DOCX 23 KB) [file 10388_2026_1200_MOESM1_ESM.docx]

**SUPPLEMENTARY MATERIAL**

**Supplementary Table 1. Summary of best objective response for participants in the Japanese subgroup with PD-L1 CPS ≥10, ESCC, and ESCC and PD-L1 CPS ≥10**

|  | **Participants with  PD-L1 CPS ≥10** | | **Participants with  ESCC** | | **Participants with ESCC and  PD-L1 CPS ≥10** | |
| --- | --- | --- | --- | --- | --- | --- |
|  | **Pembrolizumab + chemotherapy**  **n = 48** | **Placebo +** **chemotherapy**  **n = 36** | **Pembrolizumab + chemotherapy**  **n = 67** | **Placebo +** **chemotherapy**  **n = 59** | **Pembrolizumab + chemotherapy**  **n = 44** | **Placebo +** **chemotherapy**  **n = 32** |
| **ORR,^a^ % (95% CI)** | 60.4 (45.3-74.2) | 30.6 (16.3-48.1) | 56.7 (44.0-68.8) | 40.7 (28.1-54.3) | 59.1 (43.2-73.7) | 31.3 (16.1-50.0) |
| Difference, %  (95% CI) | 29.9 (8.3 to 48.6) | | 16.0 (-1.5 to 32.6) | | 27.8 (5.0 to 47.6) | |
| **BOR, n (%)** |  |  |  |  |  |  |
| CR | 3 (6.3) | 1 (2.8) | 4 (6.0) | 2 (3.4) | 3 (6.8) | 1 (3.1) |
| PR | 26 (54.2) | 10 (27.8) | 34 (50.7) | 22 (37.3) | 23 (52.3) | 9 (28.1) |
| SD | 13 (27.1) | 20 (55.6) | 22 (32.8) | 27 (45.8) | 12 (27.3) | 17 (53.1) |
| PD | 4 (8.3) | 5 (13.9) | 5 (7.5) | 8 (13.6) | 4 (9.1) | 5 (15.6) |
| Not evaluable^b^ | 2 (4.2) | 0 | 2 (3.0) | 0 | 2 (4.5) | 0 |
| **Time to response,^a^ median (range), months** | 2.1 (1.9 to 8.3) | 2.0 (1.9 to 2.3) | 2.0 (1.9 to 8.3) | 2.1 (1.9 to 4.4) | 2.1 (1.9 to 8.3) | 2.0 (1.9 to 2.3) |
| **DOR,^c^ median (range), months** | 10.4 (2.3+ to 65.9+) | 4.4 (3.5 to 17.0) | 10.4 (1.2+ to 65.9+) | 6.1 (3.5 to 22.8) | 10.5 (2.3+ to 65.9+) | 4.4 (3.5 to 17.0) |
| **Participants with extended DOR,^a,c^ %** |  |  |  |  |  |  |
| ≥54 months | 34.8 | NR | 29.9 | NR | 39.4 | NR |

Abbreviations: CR, complete response; DOR, duration of response; NR, not reached; ORR, objective response rate; PD, progressive disease; PR, partial response; RECIST v1.1, Response Evaluation Criteria in Solid Tumors, version 1.1; SD, stable disease.

“+” indicates there was no PD by the time of last disease assessment.

^a^Includes participants who experienced CR and PR.

^b^Postbaseline assessment available but not evaluable or response of CR, PR, or SD <6 weeks from randomization. ^c^From product-limit (Kaplan-Meier) method for censored data.

**Supplementary Table 2. All-cause adverse events in the Japanese subgroup**

|  | **Pembrolizumab + chemotherapy**  **n = 74** | | **Placebo +** **chemotherapy**  **n = 67** | |
| --- | --- | --- | --- | --- |
|  | **Any grade** | **Grade 3-4** | **Any grade** | **Grade 3-4** |
| **All cause AEs^a^** | **74 (100)** | **61 (82.4)** | **67 (100)** | **49 (73.1)** |
| Decreased appetite | 58 (78.4) | 4 (5.4) | 40 (59.7) | 7 (10.4) |
| Nausea | 57 (77.0) | 2 (2.7) | 43 (64.2) | 3 (4.5) |
| Neutrophil count decreased | 46 (62.2) | 30 (40.5) | 39 (58.2) | 23 (34.3) |
| Constipation | 45 (60.8) | 0 | 39 (58.2) | 0 |
| Stomatitis | 42 (56.8) | 3 (4.1) | 36 (53.7) | 2 (3.0) |
| White blood cell count decreased | 35 (47.3) | 14 (18.9) | 22 (32.8) | 6 (9.0) |
| Anemia | 31 (41.9) | 12 (16.2) | 34 (50.7) | 15 (22.4) |
| Diarrhea | 28 (37.8) | 4 (5.4) | 26 (38.8) | 2 (3.0) |
| Hiccups | 27 (36.5) | 0 | 20 (29.9) | 0 |
| Malaise | 27 (36.5) | 1 (1.4) | 25 (37.3) | 2 (3.0) |
| Fatigue | 26 (35.1) | 2 (2.7) | 11 (16.4) | 1 (1.5) |
| Blood creatinine increased | 24 (32.4) | 0 | 23 (34.3) | 1 (1.5) |
| Alopecia | 20 (27.0) | 0 | 13 (19.4) | 0 |
| Edema | 19 (25.7) | 0 | 18 (26.9) | 0 |
| Platelet count decreased | 18 (24.3) | 1 (1.4) | 14 (20.9) | 1 (1.5) |
| Dysgeusia | 17 (23.0) | 0 | 13 (19.4) | 0 |
| Peripheral sensory neuropathy | 16 (21.6) | 1 (1.4) | 14 (20.9) | 0 |
| Hyponatremia | 13 (17.6) | 11 (14.9) | 19 (28.4) | 11 (16.4) |
| Lymphocyte count decreased | 12 (16.2) | 7 (9.5) | 8 (11.9) | 2 (3.0) |
| Infusion site extravasation | 11 (14.9) | 0 | 9 (13.4) | 0 |
| Insomnia | 11 (14.9) | 0 | 8 (11.9) | 0 |
| Vomiting | 11 (14.9) | 1 (1.4) | 11 (16.4) | 0 |
| Pruritus | 10 (13.5) | 1 (1.4) | 2 (3.0) | 0 |
| Pneumonia | 9 (12.2) | 4 (5.4) | 7 (10.4) | 4 (6.0) |
| Pyrexia | 9 (12.2) | 0 | 12 (17.9) | 0 |
| Rash maculopapular | 9 (12.2) | 4 (5.4) | 0 | 0 |
| Headache | 8 (10.8) | 0 | 1 (1.5) | 0 |
| Hypokalemia | 8 (10.8) | 4 (5.4) | 5 (7.5) | 3 (4.5) |
| Nasopharyngitis | 8 (10.8) | 0 | 6 (9.0) | 0 |
| Pneumonia aspiration | 7 (9.5) | 3 (4.1) | 9 (13.4) | 4 (6.0) |
| Weight decreased | 7 (9.5) | 0 | 9 (13.4) | 1 (1.5) |
| AST increased | 6 (8.1) | 0 | 7 (10.4) | 1 (1.5) |
| Hyperkalemia | 6 (8.1) | 2 (2.7) | 12 (17.9) | 3 (4.5) |
| Hypoalbuminemia | 6 (8.1) | 1 (1.4) | 9 (13.4) | 1 (1.5) |
| Vasculitis | 6 (8.1) | 0 | 8 (11.9) | 0 |
| Rash | 6 (8.1) | 0 | 10 (14.9) | 0 |

Data are n (%).

AEs, adverse events; AST, aspartate aminotransferase.

^a^AEs with incidence of 10% or higher in any treatment group.
